# Microproteins encoded by noncanonical ORFs are a major source of tumor-specific antigens in a liver cancer patient meta-cohort

**DOI:** 10.1126/sciadv.adn3628

**Published:** 2024-07-10

**Authors:** Marta E. Camarena, Patrick Theunissen, Marta Ruiz, Jorge Ruiz-Orera, Beatriz Calvo-Serra, Robert Castelo, Carla Castro, Pablo Sarobe, Puri Fortes, Júlia Perera-Bel, M. Mar Albà

**Affiliations:** ^1^ Hospital del Mar Research Institute, Barcelona, Spain.; ^2^ Center for Applied Medical Research (CIMA), University of Navarra (UNAV), Pamplona, Spain.; ^3^ Max Delbrück Center for Molecular Medicine in the Helmholtz Association (MDC), 13125 Berlin, Germany.; ^4^ Department of Medicine and Life Sciences, Universitat Pompeu Fabra (UPF), Barcelona, Spain.; ^5^ Centro de Investigación Biomédica en Red de Enfermedades Hepáticas y Digestivas (CIBEREHD), Pamplona, Spain.; ^6^ Instituto de Investigación Sanitaria de Navarra (IdiSNA), Pamplona, Spain.; ^7^ Cancer Clinic University of Navarra (CCUN), Pamplona, Spain.; ^8^ Spanish Network for Advanced Therapies (TERAV ISCIII), Madrid, Spain.; ^9^ Catalan Institute for Research and Advanced Studies (ICREA), Barcelona, Spain.

## Abstract

The expression of tumor-specific antigens during cancer progression can trigger an immune response against the tumor. Here, we investigate if microproteins encoded by noncanonical open reading frames (ncORFs) are a relevant source of tumor-specific antigens. We analyze RNA sequencing data from 117 hepatocellular carcinoma (HCC) tumors and matched healthy tissue together with ribosome profiling and immunopeptidomics data. Combining human leukocyte antigen–epitope binding predictions and experimental validation experiments, we conclude that around 40% of the tumor-specific antigens in HCC are likely to be derived from ncORFs, including two peptides that can trigger an immune response in humanized mice. We identify a subset of 33 tumor-specific long noncoding RNAs expressing novel cancer antigens shared by more than 10% of the HCC samples analyzed, which, when combined, cover a large proportion of the patients. The results of the study open avenues for extending the range of anticancer vaccines.

## Teaser

A set of uncharacterized small proteins expressed only in tumors could be exploited to develop anticancer vaccines.

## INTRODUCTION

Immunotherapy approaches against cancer, including immune checkpoint inhibitors (ICIs) and vaccines, rely on the ability of the immune system to recognize “nonself” antigens bound to human leukocyte antigen (HLA) molecules. Such neoepitopes can originate not only from nonsynonymous mutations in the cancer genome that result in mutated peptides but also from aberrant gene expression in tumors. The first class of antigens is especially relevant in cancers associated with a large number of mutations, such as melanoma, lung cancer, or bladder cancer (*1*). Expectedly, tumor mutational burden and the number of mutated peptides with predicted affinity to HLA molecules are positively correlated with the response to ICIs (*2*, *3*).

The second class of antigens might be particularly relevant to develop therapeutic strategies for tumors that mutate less frequently, such as hepatocellular carcinoma (HCC), which represents ~90% of cases of liver cancer. Known cancer-specific antigens include the so-called cancer/testis antigens (CTAs) as well as peptides derived from reactivated human endogenous retroviruses (HERVs) (*4*). These antigens can be found in different cancer types, and they can be shared by several patients. Some of them, such as MAGE1A and NY-ESO, have been the basis of several cancer vaccines (*5*). Current limitations are the relatively low number of suitable targets with high tumor specificity and their sparse expression in cancer patient samples.

A promising approach to expand the current range of cancer-specific antigens that can be targeted by immunotherapy approaches is to consider the translation products of noncanonical open reading frames (ncORFs). These ORFs are located in sequences that are not annotated as protein coding. One well-studied example is the MELOE-1 and MELOE-2 peptides encoded by the long noncoding transcript meloe (*6*, *7*). This transcript is overexpressed in melanomas, and the encoded peptides generate a reactive T cell response (*8*). In the past few years, thousands of long noncoding RNAs (lncRNAs) containing ncORFs that are translated into microproteins have been described previously (*9*, *10*). In addition, mass spectrometry (MS) immunopeptidomics data from cancer cell lines and tumors indicate that ncORFs can generate peptides that are presented by HLA molecules (*11*–*17*). It has been reported that ncORF products can represent up to 15% of the HLA-I–bound peptides in certain tumor types (*18*), a sizable fraction that remains largely uncharacterized. In addition, they appear to give rise to HLA-I–bound peptides more frequently than standard proteins (*19*).

To be able to avoid immune self-tolerance, the ncORFs need to be expressed in a tumor-specific manner. However, due to the lack of studies comparing tumor and healthy tissues from the same set of patients, it is unclear how many of the previously reported ncORF-derived antigens are actually restricted to tumors. Thus, it is not known if peptides derived from ncORFs could be relevant as therapeutic targets. To address these questions, we have focused on tumor and matched healthy tissue sequencing data from a larger number of patients with HCC. Treatment of HCC in advanced stages remains a challenge (*20*). Because this is a type of cancer with relatively few mutations, antigens derived from tumor-specific transcripts could play a major role in driving immunogenicity. We present data supporting that ncORFs are a relevant source of tumor-specific antigens in HCC. The findings could have important implications for the development of cancer vaccines of wide applicability.

## RESULTS

### The integration of different tumor/normal matched datasets results in a large meta-cohort for the discovery of tumor-specific transcripts

**Fig. 1.**
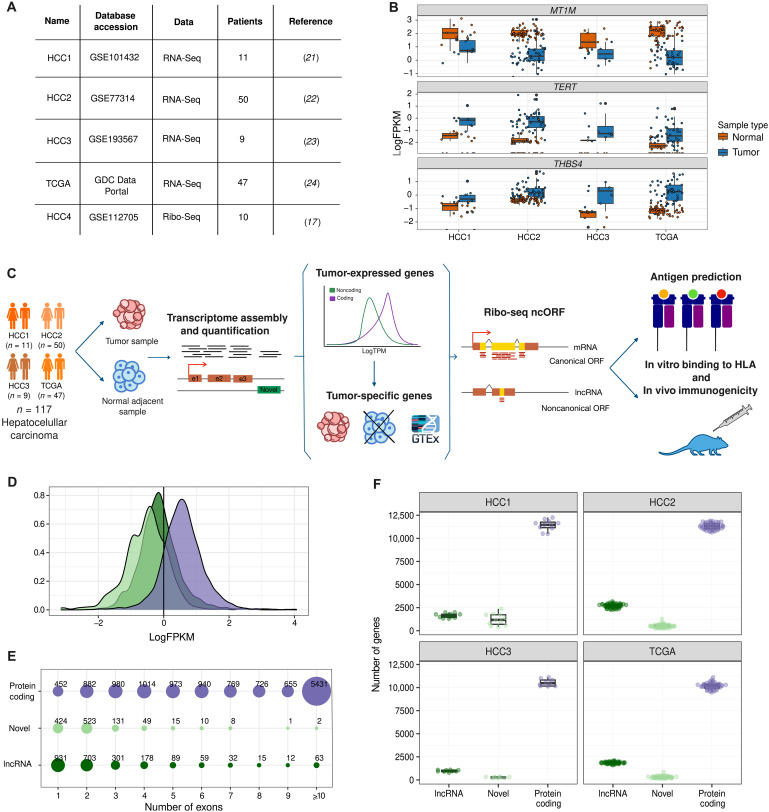
HCC transcriptome. (**A**) Datasets used. They comprise four cohorts with matched tumor-adjacent tissue RNA-Seq (HCC1 to HCC3 and TCGA) and one cohort with matched tumor-adjacent tissue Ribo-Seq data (HCC4). (**B**) Biomarkers of HCC in the four datasets. Gene expression was measured as FPKM, in both tumor and adjacent tissue samples (normal). By paired Wilcoxon signed-rank test, we confirmed that the expression of *MT1M*, *TERT*, and *THBS4* coding genes was significantly different between tumor samples and adjacent tissue following the expected trends (*MT1M* *P* value = 1.596 × 10^−19^; *TERT* *P* value = 1.578 × 10^−19^; *THBS4* *P* value = 9.941 × 10^−20^). (**C**) Main steps of the computational and experimental pipeline. We gathered RNA-Seq and Ribo-Seq data from matched tumor/normal samples. We quantified gene expression and reconstructed nonannotated transcripts. We then determined the tumor-specific transcriptome in each patient. We predicted the translation rate of lncRNAs and novel transcripts using the HCC Ribo-Seq data. We quantified tumor-specific antigens derived from ncORFs versus other sources and performed experiments to validate HLA-binding and immunogenicity. (**D**) Distribution of gene expression levels for different types of transcripts. lncRNAs and novel transcripts tended to be expressed at lower levels than protein-coding genes, although there was a considerable overlap in expression levels between the classes. The line at 1 FPKM indicates the expression cutoff used to consider a transcript as expressed. Data shown are for dataset HCC3. (**E**) Number of exons in different types of transcripts. lncRNAs and novel transcripts tended to have a lower number of exons than coding genes; the data shown are for the HCC3 dataset. (**F**) Relative abundance of different types of transcripts in tumors. Coding genes were the largest class of expressed transcripts, followed by lncRNAs and novel transcripts.

We identified four HCC patient cohorts with transcriptomics data for both tumor and adjacent normal tissue (Fig. 1A, HCC1 to HCC3 and TCGA, and table S1) (*21*–*24*). Together, this represented a meta-cohort of 117 patients. We also identified ribosome profiling (Ribo-Seq) sequencing data from an additional set of 10 HCC tumors (HCC4) (*17*). We used several previously described HCC biomarkers to validate these datasets: two genes that tend to be overexpressed in HCC—*TERT* (*24*) and *THBS4* (*25*)—and one that is usually underexpressed—*MT1M* (*26*). Consistent with these previous findings, we found that *TERT* and *THBS4 *had significantly higher expression levels in tumor than in normal matched samples in all cohorts and that *MT1M* showed the opposite tendency (Fig. 1B).

After validating the datasets with the above biomarkers, we designed a pipeline that combined different computational and experimental methods to unravel the impact of ncORFs in the generation of tumor-specific antigens in the set of 117 patients (Fig. 1C). The first steps were centered on the quantification of gene expression, the discovery of novel transcripts, and the identification of tumor-specific transcripts from tumor/normal matched RNA sequencing (RNA-Seq) data. We also predicted ncORF translation by the analysis of Ribo-Seq data and putative HLA-I–binding peptides using patient-specific HLA information (table S2). To validate the predictions, we performed in vitro HLA-peptide binding assays of a subset of the candidates as well as immunogenicity experiments in mice expressing the human HLA molecule (Fig. 1C). The analysis provided information about the quantitative relevance of different types of tumor antigens in HCC. It was also informative on the distribution of these antigens in the patient population. We identified a set of highly tumor-specific lncRNAs containing ncORFs with translation and immunopeptidomics evidence. The results are described in the next sections.

### Thousands of noncoding transcripts are expressed in HCC tumors

We used the RNA-Seq data from the four HCC cohorts to quantify the expression level of protein-coding genes and lncRNAs as well as to perform genome-guided de novo transcript assembly and identify transcripts not annotated in Ensembl (novel transcripts). LncRNAs and novel transcripts showed overall lower expression values than protein-coding genes (Fig. 1D); only those expressed above a given cutoff [fragments per kilobase million (FPKM) > 1 or FPKM > 2 depending on the dataset] were selected for further analyses (fig. S1 and tables S3 to S5). As expected, lncRNAs and novel transcripts tended to have a lower number of introns than protein-coding genes (Fig. 1E and fig. S2). We also noted that most novel transcripts, even if not annotated in Ensembl, matched entries in miTranscriptome, a gene database that contains an extended set of cancer transcripts (fig. S3) (27). Each tumor sample expressed around 10,000 to 12,000 protein-coding genes together with 2000 to 4000 noncoding transcripts (lncRNAs and novel transcripts) (Fig. 1F and fig. S4). We found that, in general, the expression of lncRNAs and novel transcripts was more patient-specific than the expression of protein-coding genes (fig. S5).

### Tumor lncRNAs are pervasively translated

**Fig. 2.**
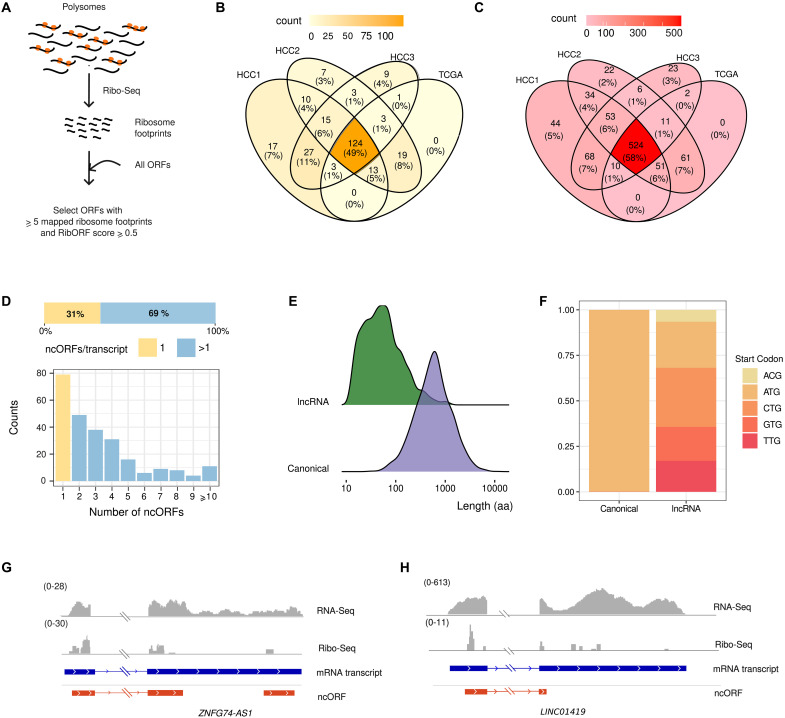
Translation of ncORFs in tumor-expressed lncRNAs. (**A**) Prediction of translated ORFS using Ribo-Seq data. From the total predicted noncanonical ORFs, we analyzed translation patterns in ncORFs with at least five mapped Ribo-Seq reads, selecting those that had a RibORF score of at least 0.5. (**B**) Comparison of lncRNAs containing ncORFs with signatures of translation from different cohorts. The intersection between the sets of translated lncRNAs in the four different transcriptomics cohorts is shown. Translated lncRNAs (124 of a total of 251) were shared across all cohorts. (**C**) Comparison of ncORFs with signatures of translation from different cohorts. The intersection between the sets of translated ncORFs shown in the different cohorts is shown. Translated ncORFs (524 of a total of 909) were shared across all cohorts. (**D**) Many lncRNAs contain several putatively translated ncORFs. The graph shows the distribution of the number of translated ncORFs per transcript. From a total of 251 lncRNAs, 79 translated one single ncORF and 172 translated more than one ncORF. (**E**) ncORFs are significantly smaller than canonical coding sequences. Comparison of the ORF length distribution of micropeptides encoded by ncORFs versus canonical ORFs, with median values of 39 and 456 amino acids (aa), respectively. Differences are significant at a *P* value of <2.2 × 10^−16^ (Kolmogorov-Smirnoff test). (**F**) Frequency of different start codons in canonical coding sequences and ncORFs. ATG as well as ACG, CTG, TTG, and GTG were considered as putative start codons. (**G**) Translation of *ZNF674-AS1*. Coverage of RNA-Seq and Ribo-Seq reads and putatively translated ORFs are indicated. The second exon of the mRNA transcript is shortened for visualization purposes. No Ribo-Seq signal was detected in the region not shown. (**H**) Translation of *LINC01419*. Coverage of RNA-Seq and Ribo-Seq reads and putatively translated ORFs are indicated.

Recent studies have shown that many lncRNAs contain ORFs that are translated into small proteins or microproteins (*9*, *10*, *28*). Here, we used Ribo-Seq data from HCC (cohort HCC4; Fig. 1A) to predict the level of translation of the previously identified tumor lncRNAs and novel transcripts (cohorts HCC1 to HCC3 and TCGA). To obtain reliable estimates, we focused on those transcripts that were widely expressed in HCC4 and at least another cohort (see Materials and Methods). In addition to ATG, we also considered near cognate codons (ACG, CTG, GTG, and TTG) as putative start sites as these codons have been shown to frequently initiate translation of ncORFs (*13*, *19*). Translation was predicted using RibORF (v1.0) (Fig. 2A and fig. S6; see Materials and Methods) (*29*). We identified 251 unique translated lncRNAs, including 124 transcripts that were common to all cohorts (Fig. 2B). A large fraction of the latter transcripts (86 of 124) had also been predicted to be translated in a study that analyzed different cancer cell lines and tumors (table S6) (*13*), which reinforced our results. Because the latter study did not include HCC data, this also implies that many of these lncRNAs are expressed in different cancer types.

The number of ncORFs for which translation was detected was 909, with 524 being common to all cohorts (Fig. 2C). Most of the transcripts contained multiple translated ncORFs (Fig. 2D). As expected, the resulting proteins tended to be smaller than canonical proteins (Fig. 2E). Translation predictions comprised ORFs initiated not only at ATG but also at alternative sites, especially CTG (Fig. 2F).

We used the Ribo-Seq data to compute a translation index for lncRNAs and novel transcripts, which we defined as the fraction of ncORF sequence predicted to be translated. In the case of lncRNAs, the translation index was 0.116. It was calculated taking into account the total percentage of translated ORFs (8.3%; table S7) as well as the fact that translated ORFs tended to be somewhat longer than nontranslated ORFs (fig. S7). The same estimation for novel transcripts resulted in a much smaller translation index (0.0053), indicating that the latter transcripts are rarely translated.

Figure 2 (G and H) shows examples of putatively translated ORFs in *ZNF674-AS1* and *LINC01419*, respectively. *ZNF674-AS1* is transcribed in antisense direction to the protein-coding gene* ZNF674 *through the use of a bidirectional promoter and low expression in tumors is associated with bad prognosis (*30*). *LINC01419* is an lncRNA that is transcribed and translated in tumor samples but not in the healthy controls.

### Tumor-specific transcripts are enriched in lncRNAs and novel transcripts

**Fig. 3.**
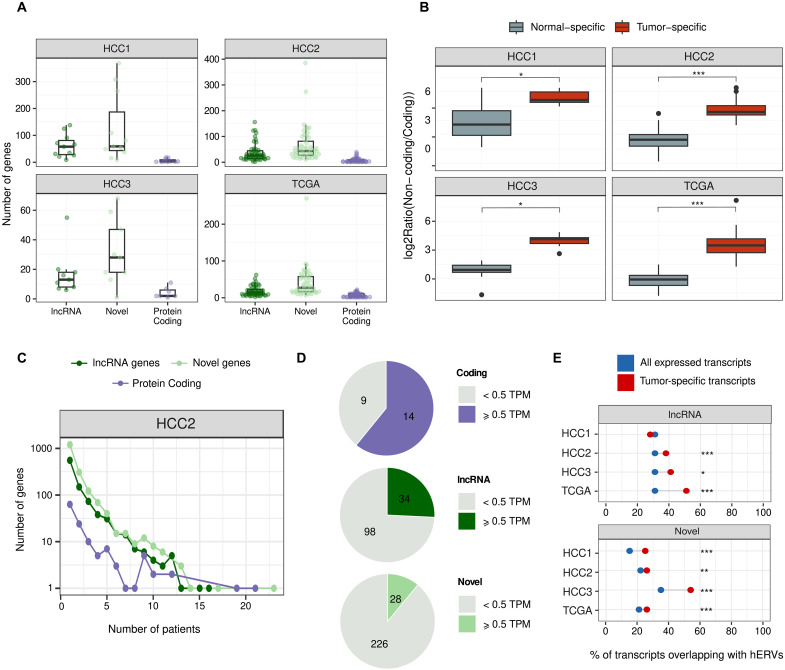
Most tumor-specific transcripts are noncoding. (**A**) lncRNAs and novel transcripts tend to be more tumor-specific than coding genes. The number of different types of transcripts per patient and cohort is shown. (**B**) Tumor-specific versus normal-specific gene expression. By paired Wilcoxon signed-rank test, we confirmed that the tumors are enriched in noncanonical genes with respect to coding ones (HCC1 *P* value = 4.883 × 10^−03^; HCC2 *P* value = 7.773 × 10^−10^; HCC3 *P* value = 7.813 × 10^−03^; TCGA *P* value = 2.469 × 10^−09^). (**C**) Shared tumor-specific transcripts. Despite the privacy of most tumor-specific transcripts, a subset is found in several patients. (**D**) Expression in testis. Proportion of tumor-specific transcripts that are also expressed in testis for different transcript types and datasets. (**E**) Proportion of lncRNA and novel transcripts overlapping HERVs. Differences between the fraction of lncRNAs overlapping HERVs in the complete transcriptome and in the tumor-specific transcriptome. Differences are significant in all cohorts except for lncRNA-HCC1 (*P* value < 0.05, Fisher’s exact test). Statistical significance is indicated as follows: ***P < 0.001, ** *P* < 0.01, *P < 0.05.

Microproteins generated from ncORFs in tumor-specific lncRNAs are a potential source of cancer antigens with immunotherapy applications, as described for some of the canonical CTAs (*31*). To determine how many of the ncORFs expressed in tumors were tumor-specific, we discarded cases that were expressed in matched healthy liver samples, Genotype-Tissue Expression (GTEx) gene expression tables for nonreproductive organs, or in a collection de novo assembled transcriptomes from diverse healthy organs (see Materials and Methods). Expression in testis was not considered an impediment as this is an immunocompromised tissue that can also express antigens of interest for anticancer vaccination. Notably, we found that, among tumor-specific transcripts, lncRNAs and novel transcripts were more numerous than protein-coding genes (Fig. 3A, fig. S8, and table S8 and S9). This was in sharp contrast with the observations for overall tumor expression, which was dominated by protein-coding transcripts (Fig. 1F).

One possibility was that the enrichment of lncRNAs and novel transcripts in the tumor-specific transcriptome could be attributed to the differences in expression levels or other factors unrelated to cancer. To assess this, we obtained the normal-specific transcriptome from each patient by subtracting the transcripts that were also expressed in the tumor. We did not find a consistent enrichment of noncoding transcripts in this transcriptome (fig. S9). As a result, the ratio between tumor-specific and normal-specific transcripts was significantly higher in noncoding transcripts than in coding ones (Fig. 3B). These results reinforced the notion that noncoding transcripts tend to be expressed in a tumor-specific manner much more frequently than coding ones.

Patient-shared tumor-specific antigens are of particular interest to develop therapies targeted at multiple patients. We inspected the number of patient tumor samples expressing the different types of tumor-specific transcripts. We found that the three types of transcripts—lncRNA, protein-coding, and novel—could be shared by a substantial fraction of the patients (Fig. 3C, fig. S10, and table S10). No single tumor-specific transcript was shared across all patients; this was in line with a previous report for CTAs, typically found in a percentage of the patient tumor samples (*31*).

Because many cancer antigens, such as the melanoma antigen genes (MAGEs), are expressed in germinal cells, we wanted to investigate if the same was true for tumor-specific noncoding transcripts. The analysis of the expression of tumor-specific protein-coding genes in testis confirmed that most of them showed significant expression in this organ (61 to 68% depending on the cohort). We also found that a subset fraction of the tumor-specific lncRNAs was expressed in testis (23 to 40%) (Fig. 3D and fig. S11). In contrast, almost no expression in testis samples was detected for the novel transcripts.

lncRNAs are known to be enriched in remnants of transposable elements (TEs) when compared to protein-coding genes (*32*). We investigated if tumor-specific lncRNAs were different from the rest of lncRNAs regarding their TE composition by inspecting their overlap with TE annotations derived from RepeatMasker (*33*). No differences were found for most TE families except for HERVs, which were significantly enriched among tumor-specific transcripts (Fig. 3E and table S11). HERVs have been reported to become activated during cancer (*34*). The demethylation of regions containing HERVs might play a role in the increased expression of HERV-containing lncRNAs.

### Many tumor-specific antigens are likely to derive from ncORFs

**Fig. 4.**
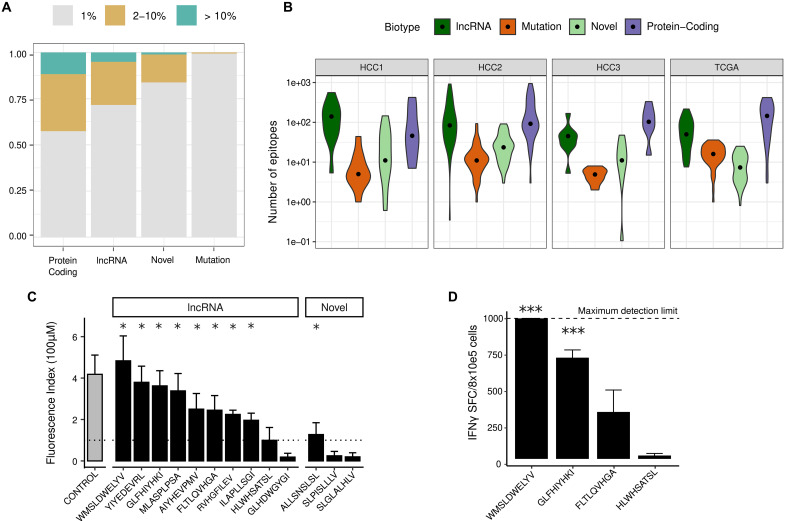
ncORFs make a substantial contribution to the HCC antigen landscape. (**A**) Proportion of shared or private predicted antigens. Antigens derived from mutations are almost all patient-specific, whereas antigens derived from tumor-specific transcripts can be shared across patients. (**B**) Predicted number of antigens per patient and dataset. Antigen load was predicted by selecting peptides with HLA-binding affinity IC_50_ < 50 nM as predicted by NetMHCpan, using patient-specific HLA allele information. For lncRNAs and novel transcripts, it was then corrected by the translation index, which is the fraction of ncORF estimated to be translated by the analysis of Ribo-Seq data. (**C**) HLA-A*02.01 binding assays for ncORFs. Binding affinity expressed as FI ± SEM for each peptide in an in vitro T2 cell binding assay. The FI value shown corresponds to the mean of two different assays (with two replicates each). A line at FI = 1 indicates the expectation under no binding. * indicates *P* value of <0.05 when comparing the values with the peptide (Wilcoxon-Mann-Whitney test). Information on the transcripts/ncORFs can be found in tables S16 to S18. CONTROL refers to a positive control (peptide 58-66 from influenza matrix protein). (**D**) IFN-γ ELISPOT assays**.** The spleens of mice immunized with four peptides were processed to measure the number of IFN-γ secreting cells (IFNγSFC). In the case of the peptide WMSLDWELYV, the measurement was >1000 IFNγSFC per 8 × 10^5^ cells in all four replicates of the experiment. Of the four peptides tested, two yielded highly significant results, WMSLDWELYV and GLFHIYHKI (***P value < 0.001, *t* test), and the other two were not significant.

It is currently unknown which is the relative contribution of ncORFs to the generation of tumor-specific antigens when compared to other antigen sources in HCC. To estimate this, we first used NetMHCpan to predict all possible 9-mer peptides with strong binding affinity to HLA-I [median inhibitory concentration (IC_50_) < 50 nM] for all tumor-specific ncORFs and coding sequences as well as for peptides containing somatic mutations. The latter was obtained by performing variant calling directly from the transcriptomics sequencing data (HCC1, HCC2, and HCC3) or from already available mutation data (TCGA). Because a peptide’s HLA binding affinity depends on the specific HLA allele, we inferred the HLA alleles of each patient using the RNA-Seq data and then performed predictions by HLA type. The proportion of strong binders (IC_50_ < 50 nM) among all possible peptides was similar for the different types of putative identified cancer-specific antigens (8.7, 8.6, and 9.7% for lncRNA, novel, and protein-coding, respectively) and slightly lower for mutated peptides (6.5%). Putative HLA-I binders derived from mutations were essentially private (99.4%) (Fig. 4A and tables S12 and S13). In contrast, an important fraction of the other tumor-specific antigens were shared across different patients. In the case of protein-coding genes (CTAs), the fraction of predicted HLA-I binders present in more than one patient was 42.6%. The equivalent figure for lncRNAs and novel transcripts was 28.4 and 16.2%, respectively.

However, what do these observations imply at the level of individual patients? As we wanted to focus on nonself-peptides, we first identified and discarded any predicted strong binders matching other proteins/ORFs that were not tumor-specific. This eliminated 20 to 30% of the strong binders located in protein-coding genes but only 1 to 2% of those in lncRNAs and novel transcripts (table S14). This means that, in general, peptides derived from ncORFs should not generate toxicities due to the cross-reaction with other proteins if used as vaccines. Next, we multiplied the initial number of predicted strong binders in each of the patients by the translation index (0.116 for lncRNAs and 0.005 for novel transcripts). This index represents the overall fraction of the ncORF sequence space that is expected to be translated according to our previous analysis of Ribo-Seq data. The two most important contributors to the generation of antigens were tumor-specific lncRNAs and protein-coding genes (median *n* = 45 to 140 for lncRNAs and *n* = 43 to 106 for protein-coding genes, depending on the dataset; Fig. 4B and table S15). In contrast, mutated peptides generated a relatively small number of predicted antigens (median *n* = 5 to 16, depending on the dataset). In the case of novel transcripts, the low rate of translation meant that the estimates of the number of generated antigens were also low (median *n* = 7 to 23, depending on the dataset). The average relative contribution of the different types to tumor-specific antigens, considering all 117 patients, was 49% protein-coding genes, 39% lncRNAs, 7% novel transcripts, and 5% mutations.

### Most predicted strong HLA-I binders can be experimentally validated

To assess the reliability of the HLA binding predictions, we performed in vitro testing of 13 ncORF-derived peptides with high binding affinity for HLA-A*02:01 using HLA-A*02:01^+^ T2 cells. The assay measures the peptide’s ability to bind and stabilize HLA class I molecules, which are otherwise rapidly degraded in its normal peptide-unbound form. We selected five peptides derived from ncORFs in the top most shared tumor-specific lncRNAs (>22% of the patients) and five peptides from less frequently occurring lncRNAs. We also included three peptides derived from ncORFs in novel transcripts. We found that 8 of 10 peptides derived from ncORFs encoded by lncRNAs and 1 of 3 peptides derived from ncORFs encoded by novel transcripts showed significant binding to the HLA molecule [measured as fluorescence index (FI) in Fig. 4C; tables S16 and S17]. The 50% maximal binding capacity for these peptides was in the range of 15 to 70 μM (table S16). Overall, 9 of 13 tested computational predictions were experimentally validated, which provides an estimate of around 70% of the predictions being actual HLA binders.

Four of the peptides with high predicted HLA-A*02:01 binding affinity were injected in HDD-DR1 mice, which contains genes encoding HLA-A*02:01 and HLA-DRB1*01, to test if they could elicit an immune response (Fig. 4D and table S18). Mice were immunized twice with the peptides, and 14 days after the initial immunization, T cell response was measured through interferon-γ (IFN-γ) enzyme-linked immunospot assay ELISPOT assay. Peptide immunization resulted in strong IFN-γ signal in two cases (WMSLDWELYV in AC079466.1 and GLFHIYHKI in AC098820.3), indicating peptide immunogenicity. These responses were specifically induced by the peptides because splenocytes from mice immunized with the unrelated HLA-A*02:01–restricted influenza matrix 58 to 66 peptide did not recognize any of them (table S18). Moreover, according to their binding capacity to major histocompatibility complex (MHC) class I molecules, responses were mediated by CD8^+^ but not CD4^+^ T cells (fig. S12 and table S18).

### Shared tumor-specific lncRNAs are expressed in different groups of patients

**Fig. 5.**
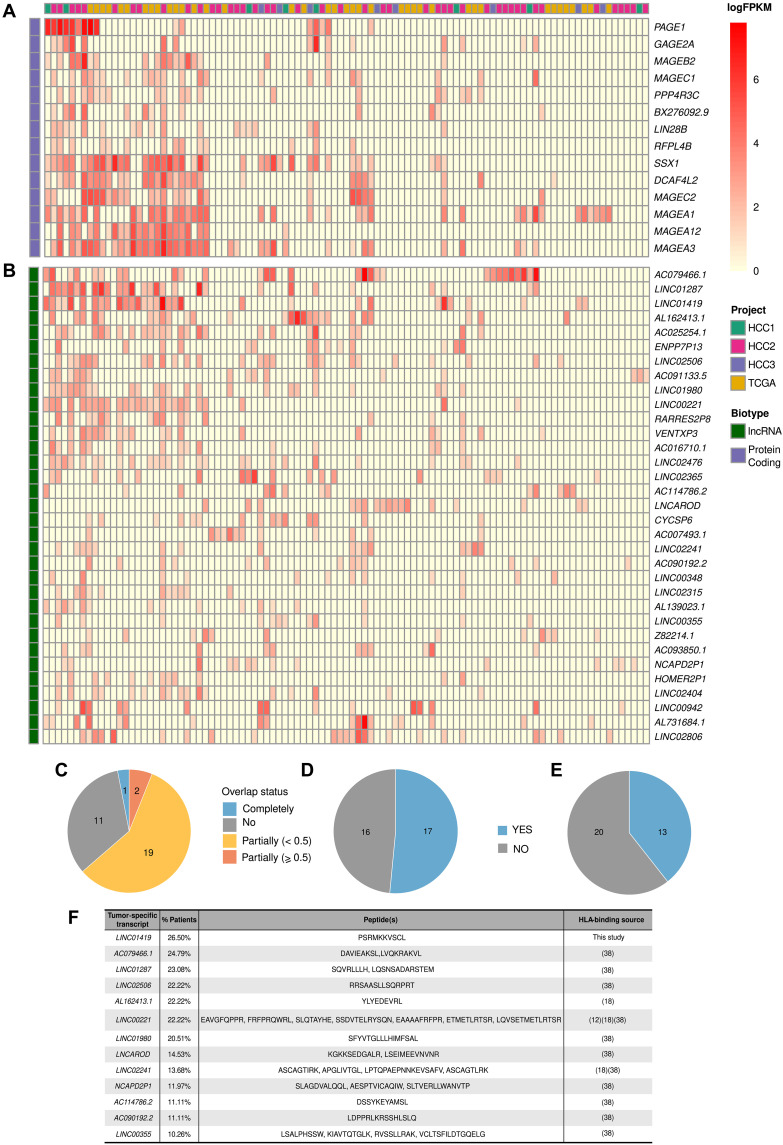
Tumor-specific transcripts shared by more than 10% of the patients. Only genes that were tumor-specific in >10% of the HCC tumor samples, expressed at more than 5 FPKM in at least one sample and expressed in less than 1% of the normal liver samples (FPKM cutoff = 1), were considered. The intensity of the color in the cell reflects the level of expression (minimum of 1 FPKM). (**A**) Protein-coding transcripts. Number of transcripts: 14. The transcripts tend to cluster in the leftmost group of patients. (**B**) lncRNAs. Number of transcripts: 33. The transcripts are scattered across different patients. (**C**) Overlap with HERVs for this set of lncRNAs. Overlap is based on genomic coordinates. (**D**) Proportion of lncRNAs with detected translated ncORFs. Prediction of ncORF translation was performed using Ribo-Seq data from a different HCC cohort of 10 patients (HCC4). (**E**) Proportion of lncRNAs containing ncORFs with immunopeptidomics evidence in different cancer datasets. (**F**) List of lncRNAs and peptides with immunopeptidomics evidence. The source of the data is indicated.

The analysis of the distribution of tumor-specific antigens across patient tumor samples is important to determine if they tend to cluster in the same group of patients or if they instead show a sparse distribution. If the second is true, potential multipeptide vaccines could be effective in a larger number of patients. To examine this question, we selected transcripts shared by more than 10% of the patients that were expressed at relatively high levels [>5 fragments per kilobase per million mapped reads (FPKM) in at least one sample] and showed high tumor-specificity (expressed in less than 1% of the tumor adjacent samples considering all 117 patients) (table S19). This resulted in 14 protein-coding genes and 33 lncRNAs (Fig. 5A). Analysis of RNA-Seq data from an unrelated cohort of 161 HCC tumor samples (*35*) showed that most of them (68%) were also expressed in more than 10% of the patients in the independent cohort (fig. S13). We also tested the expression of these transcripts in the thymus using previously published data (*11*, *36*, *37*). Expression in this organ induces central immune tolerance to self-peptides. No relevant expression in thymic cells was observed for any of the transcripts, except for *LINC02315* (table S20).

Most protein-coding genes in this set tended to cluster in the same group of patients, suggesting similar gene expression activation mechanisms (Fig. 5A). Instead, lncRNAs tended to be more scattered across patients (Fig. 5B). Accordingly, gene expression correlation values between pairs of protein-coding genes tended to be higher than between pairs of lncRNAs (fig. S14). In particular, the average correlation between any two protein-coding genes was 0.44, whereas for lncRNAs, it was 0.19. Furthermore, most protein-coding genes (11 of 14) were located in the X chromosome, whereas lncRNAs were dispersed across different chromosomes (table S21). In line with the previous observation that tumor-specific lncRNAs were enriched in HERV-derived sequences, we found that 21 of the 33 lncRNAs showed some degree of overlap with HERVs (Fig. 5C and table S22). This points to a possible link between suppression of the silencing of HERV-containing genomic regions in tumors and the activation of normally silent transcripts.

### Shared tumor-specific lncRNAs are frequently translated and can produce HLA-bound peptides

We detected signatures of translation in more than half of the lncRNAs in this subset (17 of 33) using Ribo-Seq data from the HCC4 cohort and the same procedure as previously described (Fig. 5D and table S22). Furthermore, 12 of these lncRNAs encoded HLA-bound peptides according to the results of different cancer immunopeptidomics studies (*12*, *13*, *18*, *38*). We uncovered an additional one, *LINC01419*, by the analysis of raw immunopeptidomics data from HCC (Fig. 5, E and F, and tables S22 and S23) (*39*). Because the first studies did not include HCC tumors, this indicates that the lncRNAs can be expressed in other cancer types as well. Two of the lncRNAs were supported by more than one study. One of them was *LINC00221*, reported by Erhard *et al.* (*18*), Chong *et al.* (*12*), and Cai *et al.* (*38*) (IEAtlas), and the other one was *LINC02241*, by Erhard *et al.* (*18*) and Cai *et al.* (*38*) (IEAtlas). These results strongly suggest that lncRNAs are a frequent source of shared tumor-specific antigens.

There were 10 lncRNAs that were expressed in more than 20% of the tumor samples (*LINC01980*, *AC025254.1*, *LINC02806*, *LINC02476*, *LINC02506*, *AL162413.1*, *LINC00221*, *LINC01287*, *AC079466.1*, and *LINC01419*), compared to five protein-coding genes (*MAGEA1*, *MAGEA3*, *SSX1*, *DCAF4L2*, and *MAGEC2*). We found evidence of ncORF translation for eight of them (table S22). In addition, five of them encoded peptides that are likely to bind to HLA-I according to the analysis of immunopeptidomics data (*AC079466.1*, *AL162413.1*, *LINC00221*, *LINC02506*, and *LINC01419*) (Fig. 5F). The lncRNA *LINC00221* has been previously shown to have an antitumor effect by inhibiting HCC cell growth, migration, and invasion (*40*). In contrast, *AC079466.1* has been associated with poor prognosis in HCC (*41*). One of the peptides encoded by *AC079466.1* showed high HLA binding affinity in the experiments with T2 cells as well as immunogenicity in HLA transgenic mice (Fig. 4D).

## DISCUSSION

In this work, we have shown that microproteins translated from ncORFs can substantially expand the number of tumor-specific antigens over traditional sources, providing additional targets for immunotherapy. We have designed a pipeline that integrates different sources of data from tumors and matched healthy tissue to characterize the tumor-antigen landscape, which we have then applied to a meta-cohort of 117 patients with HCC. This pipeline successfully identifies known CTAs as relevant in HCC, including the melanoma-associated antigen (MAGE) family, CTA G (GAGE), or the synovial sarcoma X breakpoint 1 antigen (SSX1) (*31*, *42*). However, it also identifies ncORFs in lncRNAs as important players in defining the tumor antigen landscape. In contrast to canonical cancer antigens, the lncRNAs do not show a strong clustering in the same subset of patients. This means that they could potentially be used to target a larger number of patients.

We studied both lncRNAs and nonannotated, novel transcripts in the tumor as a possible source of tumor antigens. Using Ribo-Seq data from patients with HCC, we found that translated ncORFs concentrated in lncRNAs, whereas almost no translation signatures were detected in novel transcripts. Ribo-Seq has been instrumental to uncover the translation of many small ORFs that had not been initially annotated as coding (*10*, *27*, *43*). These studies have also revealed that ncORFs in lncRNAs and mRNA untranslated regions can initiate translation from codons other than ATG. Here, we leveraged HCC Ribo-Seq data to infer the rate of translation of the total ncORFome in HCC tumors as well as to detect translated ncORFs in a subset of patient-shared tumor-specific lncRNAs. Our results are in line with the notion that many lncRNAs contain translated ncORFs. The median length of the microproteins encoded by ncORFs was 39 amino acids, similar to that described in a large set of Ribo-Seq ORFs recently cataloged in GENCODE (average of 44 amino acids) (*10*).

We also gathered evidence that ncORFs can generate HLA-bound peptides using information from previous studies (*17*, *18*, *20*, *43*) as well as from the analysis of MS-based proteogenomic data of HLA-bound peptides from HCC samples (*39*). Our results are in line with a previous study that reported that proteins translated from ncORFs represent at least 10% of the HLA-I presented peptides (*16*). It was also shown that cryptic proteins derived from ncORFs are particularly efficient at generating HLA-I peptides (*21*). One explanation is that, while many of these proteins are likely to be highly unstable in the cytoplasm, they last for much longer when bound to HLA-I (*44*).

Previous studies on cancer lncRNAs have mainly focused on lncRNA tumor overexpression (*45*, *46*). It has been shown that some of the overexpressed lncRNAs have oncogenic activities, such as promoting cellular proliferation and interfering with epithelial-to-mesenchymal transition regulators (*46*). The activity can be mediated by the RNA molecule itself or by encoded microproteins. One example of the latter class is *LINC00998*. This lncRNA codes for the SMIM30 micropeptide, which activates the mitogen-activated protein kinase pathway (*47*). In our study, we instead focused on lncRNAs that are expressed in a tumor-specific manner as they are likely to be more relevant for cancer immunotherapy.

Although the functions of most of the identified tumor-specific lncRNAs remain uncharacterized, several of them have been previously suggested to have roles in cancer. One example is* LINC02241*, which we found expressed in 13.6% of the HCC samples and has been associated with poor prognosis in colorectal cancer (*48*). Another example was *AL162413.1* (22.2% of HCC samples), described as a potential biomarker of oral squamous cell carcinoma (*49*). Last, *LNCAROD*, found in 14.5% of HCC samples, has been associated with attenuation of cell proliferation, whereas the opposite effect is observed when the gene is overexpressed (*50*). These three lncRNAs had been reported to encode HLA-bound peptides in other cancer cell types. This highlights their pervasiveness in tumors of different origins and their potential utility for pan-cancer therapies.

Epigenetic dysregulation of the cancer genome leads to the expression of genomic regions that are generally silent (*51*). For example, it was shown that the promoter of the polycistronic transcript meloe, which encodes the MELOE-1 and MELOE-2 cancer-specific peptides, is hypomethylated in melanoma (*52*). HERV sequences cover about 8% of the genome and can become reactivated in cancer (*53*). We found an enrichment of HERV sequences among tumor-specific transcripts, suggesting that they might facilitate the expression of cancer-specific lncRNAs. HERV signatures have been shown to predict immunotherapy response in clear cell renal cell carcinoma (*54*), suggesting a link between transcriptional activation of HERV-containing regions and tumor antigen production. Changes in transcription factor activity or even the formation of novel transcription factors could also have an effect. One known case is the formation of a chimeric transcription factor in Ewing sarcoma, which leads to the expression of a different set of spliced and polyadenylated transcripts; some of which can translate peptides (*55*).

For tumor antigens to be of therapeutic usefulness, they must be capable of triggering an immune response against the tumor. MAGEA1 was the first of a series of CTAs, which have been shown to be recognized by cytolytic T lymphocytes (*56*). Several of these antigens have, since then, been used to develop vaccines, with some having shown clinical benefits (*4*). However, the clinical trials have also revealed that caution needs to be taken to avoid toxicities, for example, induced by the cross-reactivity between several MAGE-A family members; not all of which are expressed in a tumor-specific manner (*57*). These problems are not expected for potential vaccines based on ncORFs as lncRNAs do not cluster into families (*58*). Consistently, we found nearly no matches between predicted strong binders encoded by ncORFs in lncRNAs and nontumor-specific proteins, whereas the same was not true for CTAs. We observed that, similar to canonical cancer antigens, lncRNAs showed expression in some, but not all, patients. The difference was that they were more diverse in their distribution patterns, which means that they could potentially be used to target groups of patients that would not be targetable with classical CTAs.

We tested four ncORF-derived peptides in mice transgenic for HLA-A*02:01 and found that two of them could generate a significant immune response involving CD8^+^ T cells. Additional support for CD8^+^ T cell responses induced by lncRNA peptides has been gathered in murine tumors (*59*). Further work will be required to test if the peptides are immunogenic in humans, but several examples have been reported in the literature that involve ncORFs. One previously described case is MELOE-1, encoded by a noncoding transcript and involved in T cell transfer efficiency (*6*). Other examples include peptides arising from the tumor-specific expression of intronic regions or alternative frames (*60*, *61*). More recently, noncanonical splicing junctions between exons and TEs have also been shown to be a source of immunogenic antigens in cancer by stimulating human T cell populations (*62*).

In the study, we combined data from different sources, which poses some limitations. For example, we used Ribo-Seq data from a different HCC cohort than the RNA-Seq data, and this decreased our ability to detect translation in transcripts that were highly patient-specific. We also used immunopeptidomics data from cancer types other than HCC to further support HLA binding of ncORF-derived peptides. In future studies, using MS data from the same tumor/matched tissue should provide more accurate estimations of the number of HLA-bound peptides that are tumor-specific. At the same time, it would allow testing if ncORFs, which encode proteins that are potentially more unstable, result in a disproportionately large number of HLA-bound peptides in HCC, as previously shown for lymphoma (*19*). For mice immunized with two different ncORF-derived peptides, we could observe an immune response mediated by CD8^+^ T cells. As a limitation of the current study, we did not show that CD8^+^ T cells are activated by HLA-A2^+^ cells expressing the source protein as a transgene or recombinant virus. In addition, we did not show that the peptides are immunogenic in humans. Demonstrating immunogenicity in T cells from patients expressing the ncORF candidates would be a necessary step to guide any future vaccine development.

In summary, our study has investigated the prevalence of ncORFs in a composite large cohort of tumor/matched HCC samples, revealing that ncORF-derived peptides can be highly tumor-specific, patient-shared, and presented by HLA molecules. The analysis has identified several promising candidates that might be involved in tumorigenesis and/or be capable of activating T cell responses after vaccination or blockade by checkpoint inhibitors. This study encourages research on ncORFs in other cancer-types and opens possible avenues for treatment.

## MATERIALS AND METHODS

### Preprocessing of raw sequencing data

We obtained RNA-Seq data containing HCC tumor/normal paired data from Gene Expression Omnibus (GEO) entries GSE101432 (*21*), GSE77314 (*63*), GSE193567 (*23*), and GSE112705 (*17*) as well as from TCGA (*24*). In the case of GSE112705, we also downloaded and analyzed Ribo-Seq data. These datasets were named HCC1, HCC2, HCC3, HCC4, and TCGA, respectively. The sequencing reads were for total RNA except for TCGA, which was polyA^+^ RNA. Files of raw reads were downloaded from the GEO database, and the Sequence Read Archive (SRA) Toolkit (v2.9.2) (https://trace.ncbi.nlm.nih.gov/Traces/sra/sra.cgi?view=software) was used to convert SRA to FASTQ format. For TCGA data, files with mapped reads (bam files) were downloaded from the Genomic Data Commons Data Portal (*64*) and reverted to FASTQ format using SamToFastq from Picard Toolkit (v2.25.1) (http://broadinstitute.github.io/picard/). RNA-Seq reads were quality assessed using both FastQC (v0.11.5) and FastQScreen (v0.14.0) software (*65*). All selected samples passed the quality control. The Cutadapt (v.2.1) program (*66*) was used to trim 3′ adapters from the raw reads with -O 5 -q 30 -m 26 parameters. Sequencing reads were aligned to the human reference genome GRCh38/p13 using two-pass alignment with STAR (v2.7.1) (*67*) to improve the quantification of not yet defined splice junctions. Only uniquely mapped reads were considered.

### De novo transcript assembly

We assembled the transcriptome of each tumor/normal sample separately with StringTie (v2.0) (*68*) in a conservative mode, using GENCODE annotation version 38 as the reference annotation file. To recover nonannotated transcripts, we selected those that did not overlap with any transcript in the human reference annotation using BEDTools (v2.2.1) (*69*). These transcripts were termed novel. Transcriptome assembly worked well in all cohorts except for one patient sample from HCC1, which appeared to have an abnormally high number of transcripts. This outlier was removed from further analysis.

In datasets HCC1 and HCC3, with strand-specific RNA-Seq data, novel transcripts had a known orientation (mapped to the plus or minus genomic strand) and a similar number of exons distribution than annotated lncRNAs. In contrast, in datasets HCC2 and TCGA, which were not strand-specific, the vast majority of these transcripts corresponded to single exon genes, and their orientation was not known. In this case, we imputed the orientation from miTranscriptome transcripts with overlapping genomic coordinates. If no matches in miTranscriptome could be found, then the transcripts were discarded. Neither transcripts shorter than 300 bp nor those longer than the longest annotated tumor lncRNA (*KCNQ1OT1*, 91666 nucleotides) were considered for further analysis.

### Transcript expression quantification

For each patient, we built a complete transcriptome by merging the annotated genes with the transcripts obtained by de novo transcript assembly and not matching any annotated genes. In the case of annotated genes, we considered coding and lncRNAs and kept the longest transcript per gene. lncRNAs included the class “processed pseudogenes.” To quantify gene expression, we used featureCounts (*70*), from the Subread package (v2.0.3), in stranded mode whenever possible. Next, we converted the counts (uniquely mapped reads) to FPKM. Transcripts with expression values lower than 1 FPKM were not considered for downstream analysis. In the case of HCC1 dataset, this cutoff was increased to 2 FPKM because lncRNAs/novel transcripts tended to have higher expression values in general. Using the 2 FPKM cutoff ensured that a similar proportion of lncRNAs/novel transcripts was recovered in this dataset when compared to the other ones.

### Merging novel transcripts from different patients

Gffcompare software (*71*) was used to merge novel transcripts from different samples on the basis of overlapping genomic coordinates and obtained a nonredundant set of representative transcripts. This step was necessary because the same transcript might be reconstructed in slightly different ways in different samples, resulting in different but overlapping genomic coordinates. Each representative transcript had a unique identifier, which we used to track the transcript across patients. We run BLASTN (v2.11) (*72*) with default parameters to detect possible homology between the representative transcripts and ribosomal RNA (rRNA) genes. Those that had significant sequence homology with rRNA genes were discarded (*E* value < 10^−3^).

### Prediction of translated ORFs

We defined ncORFs as starting with ATG, ACG, CTG, GTG, or TTG and ending with a stop codon. The ncORFs had a minimum length of 30 nucleotides. When two ncORFs overlapped in the same frame, we selected the longest one. For protein-coding genes, only the annotated coding sequence was considered. Translation was predicted using RibORF (v1.0) (*29*), which generates a score by combining the three nucleotide periodicity and homogeneity of the signal along the ORF. To increase sensitivity, we first merged the reads from 10 Ribo-Seq tumor samples (HCC4). We obtained a total of 99 million mapped Ribo-Seq reads. The reads had an average periodicity (proportion of reads in the correct frame) over 0.5 (e.g., 0.58 and 0.51 for 28- and 29-bp sequences, respectively). A minimum of five footprints and a RibORF score of at least 0.5 were the criteria used to classify any ncORF as translated. From the total set of putatively translated ncORFs, we eliminated those overlapping in the same frame, keeping only the longest one. We ended up with a nonredundant list of translated ncORFs per dataset.

Many of the lncRNAs and novel transcripts detected in the 117 HCC tumor samples are patient-specific or restricted to few patients. Because the Ribo-Seq data are for a different cohort of 10 HCC samples, we could expect low sensitivity. To obtain reliable estimates of the level of translation of lncRNAs and novel transcripts, we focused on those expressed in at least 90% of the patients in the cohort of interest as well as in the cohort with Ribo-Seq data. In the latter cohort, expression was determined using the available RNA-Seq data and a cutoff of 1 FPKM. We also used the Ribo-Seq data to investigate the translation of a subset of tumor-specific transcripts expressed in more than 10% of the individuals of the 117 HCC meta-cohorts.

### Tumor-specific gene subset

An expression cutoff value of >1 and <0.1 FPKM in tumor and adjacent normal samples, respectively, was established to select transcripts expressed only in the tumor sample in each of the patients. In the case of the HCC1 dataset, the cutoff was >2 and <0.2 FPKM, respectively, due to overall higher expression levels of noncoding transcripts (fig. S1). We also collected expression data from the Genotype-Tissue Expression (GTEx) project (*73*), which includes RNA-Seq experiments from a wide spectrum of body tissues. The data were used to discard genes with a median expression higher than 0.5 TPM in any nonreproductive tissue. Expression in testis and/or ovary was not considered because germinal cells do not express HLA molecules, and thus no antigens can be detected by the immune system. In the case of novel transcripts, which were not represented in GTEx, we used de novo transcript reconstructions using publicly available RNA-Seq data from a range of human tissues (brain, cerebellum, heart, kidney, liver, and testis) (*74*). Novel transcripts with a median expression higher than 0.5 TPM in this set of healthy tissues (with the exception of testis) were removed as well.

### Analysis of noncoding transcripts overlapping TE annotations

We examined the genomic overlap between lncRNA and novel transcripts on one hand and TE sequences on the other. We sourced TE annotations from the UCSC Genome Browser’s RepeatMasker track for the GRCh38/hg38 genome assembly (http://genome.ucsc.edu/) (*75*). Then, we removed low-complexity regions, simple repeats, satellites, rRNA, scRNA (small conditional RNA), snRNA (small nuclear RNA), srpRNA (signal recognition particle RNA), and tRNA to only keep TE instances. Moreover, we only kept TEs with known strand information (i.e., features with a strand different than “+” or “−” were discarded). To identify which tumor-specific lncRNA/novel transcripts were overlapped by TEs, we used the “findOverlaps” function from the “GenomicRanges” package (*76*), requiring a minimum overlap of 1 bp on the same strand. Next, we computed the fraction of lncRNA/novel transcript length occupied by TEs. To do so, we accounted for the possibility of two or more TEs overlapping between them while contained in the lncRNA/novel transcript, avoiding counting this TE overlapping length twice. We then compared the frequencies of different classes of TE elements [LINEs (long interspersed nuclear elements), SINEs (short interspersed nuclear elements), retrotransposons, HERV, and RNA) between tumor-expressed and tumor-specific noncoding transcripts. Only HERVs showed a significant enrichment in the tumor-specific subset of transcripts that was consistent across HCC cohorts.

### Identification of potential HLA-I–bound peptides

We used optitype from the nf-core/hlatyping pipeline (https://nf-co.re/hlatyping) and arcasHLA (*77*) to determine patients’ four-digit HLA-I. Next, netMHCpan 4.1 (*78*) was used to predict the potential immunogenicity of the previously identified sequences with coding potential. We derived all possible 9-mer peptides from canonical protein sequences and noncanonical ORFs and selected those with predicted IC_50_ < 50 nM (concentration that inhibits 50% binding of the fluorescein-labeled reference peptide) as strong HLA-I binders.

### Variant calling

For HCC1, HCC2, and HCC3 datasets, we used GATK4 best practices pipeline (*79*) described for variant calling in somatic RNA-Seq data with tumor and matched normal samples. Mutect2 (*80*) was used to detect single nucleotide variant (SNVs) that were later filtered according to standard quality metrics. For TCGA, we used previously defined somatic mutations (*24*). Only mutations from genes expressed in the patient sample were considered. Under both conditions, we required a minimum of total depth of coverage of >10 and a minimum of three reads supporting the alternative variant to obtain high-confidence SNVs. The functional annotation of the identified somatic mutations was done with Ensembl Variant Effect Predictor tool (v.98) (*81*), and only those whose consequence is missense (change of amino acid) were maintained. We identified potential neoantigens arising from the mutations in the same manner as for tumor-specific translated products, using a sliding window around the mutated amino acid.

### HLA binding studies

Potential epitopes binding to HLA-A*02:01 were predicted in silico with NetMHCpan 4.1 (*78*) from 9-mers derived from ncORFs located in tumor-specific noncoding transcripts. The selected 9-mers had a range of predicted HLA-A*02:01 affinities between 8.04 and 68.04 nM and were found in 13 different lncRNAs and 3 novel transcripts. We prioritized lncRNAs found in a wide range of patients (9 to 31 patients). For HLA-A*02:01 binding assays, peptides were synthesized with a purity of ≥80% at GeneCust. HLA-A*02:01^+^ T2 cells were used to determine peptide binding to HLA-A*02:01 molecules. Cells (2.5 × 10^5^ per well) were cultured in 96-well microplates with decreasing concentrations of the corresponding peptide and incubated overnight at 37°C. Samples were then incubated with Beriglobin (800 μg/ml) and stained with fluorescein isothiocyanate (FITC)–labeled anti–HLA-A*02 (GeneTex) (2 mg/ml; 15 min at room temperature), and mean fluorescence intensity (MFI) was determined by flow cytometry. Peptide 58-66 from influenza M protein was used as a positive control. Peptide binding was expressed as FI using the following formula: (MFI with peptide − MFI without peptide)/MFI without peptide. We performed two independent experiments for each peptide, each time taking two measurements.

### Immunization experiments

Eight-week-old female HHD-DR1 mice, transgenic for human HLA-A*02:01 molecules (*82*), were used. After study approval by the ethics review committee (reference no. 036-21), mice were bred and housed under pathogen-free conditions in the animal facility of the Center for Applied Medical Research (CIMA). Mice (*n* = 4 per group) were immunized with peptides (100 nmol per peptide), polyinosinic:polycytidylic acid (50 μg per mouse), and αCD40 (50 μg per mouse). The peptides and adjuvants were administered simultaneously via subcutaneous injection of 100 μl of the mixture resuspended in phosphate-buffered saline. Mice received a boost on day 7 and were euthanized on day 14.

### IFN-γ ELISPOT

The spleens of immunized mice were processed to measure the number of IFN-γ secreting cells. Splenocytes (8 × 10^5^ cells per well) were stimulated for 24 hours with peptides (10 μg/ml). The number of IFN-γ secreting cells was quantified by ImmunoSpot automated counter (Cellular Technology Limited) using the Spot 3 CTL CellCounting software.

### IFN-γ detection by flow cytometry

Splenocytes were stimulated with the peptides (10 μM) in the presence of GolgiStop and GolgiPlug (BD Biosciences). Four hours later, cells were surface stained with the following antibodies: CD3ε-Percp-Cy5 (145-2 C11), CD4-FITC (RM4-5), and CD8-BV421 (53-6.7) from BioLegend. Next, cells were fixed and permeabilized using BD Cytofix/Cytoperm Fixation/Permeablization Kit and intracellularly stained with IFNγ-PE (XMG1.2) antibodies. Samples were acquired with a Cytoflex (Beckman Coulter) flow cytometer and were analyzed using FlowJo software (Tree Star).

### Identification of frequently occurring tumor-specific transcripts

We selected transcripts that were tumor-specific in at least 10% of the patients (12 or more of 117), with an expression value higher than 5 FPKM in at least one patient and with a very high overall tumor-specificity. The latter was defined as expression in less than 1% of the 117 normal adjacent normal samples using an expression cutoff of 1 FPKM for HCC2, HCC3, and TCGA or 2 FPKM for HCC1. We identified 14 protein-coding genes and 33 lncRNAs that met these requirements. To validate these findings, we downloaded the normalized expression data from an external dataset with 161 tumor samples from patients with HCC from the International Cancer Genome Consortium (ICGC) data portal (http://dcc.icgc.org/; release 28). For the highly frequent tumor-specific genes, we measured the proportion of tumor samples that were expressing each gene. We used R (version 4.1.2) to measure the correlation between the percentages of patients expressing each candidate gene per study. We also analyzed the expression of this subset of tumor-specific transcripts in the thymus using 30 publicly available thymic epithelial cell (TEC) samples from two GEO entries: GSE127825 (*11*, *37*) and GSE201719 (*36*) (table S25). TEC samples were aligned to the genome and quantified using the abovementioned pipeline. Transcripts with a median expression higher than 0.5 FPKM were considered to be expressed in thymus.

### Immunopeptidomics data

We identified any matches between the HCC tumor-specific lncRNAs identified in this study and the lncRNAs with ncORFs encoding HLA-bound peptides as detected by several cancer cell immunopeptidomics data studies (table S23). The Ensembl identifiers were used to identify the matches. A brief description of the data from these studies follows. Chong *et al.* (*12*) performed MS-based proteogenomics to identify HLA-bound peptides in seven patient-derived melanoma cell lines and two pairs of lung cancer samples. We obtained the list of all peptide-spectrum matches (PSMs) for all noncanonical peptides binding to HLA with a PSM false discovery rate (FDR) of 3% in their supplementary table 3. Ouspenskaia *et al.* (*13*) used Spectrum Mill to evaluate immunopeptidomics, focusing on the contribution of translated ncORFs to the MHC-I repertoire across several cancer types, including melanoma, glioblastoma, and chronic lymphocytic leukemia. Their analysis comprises 92 HLA alleles expressed in B721.221 cells, using a global FDR of 1% and an FDR of 4.6% specifically for ncORF peptides. Results are taken from their supplementary table 8. Erhard *et al.* (*18*) introduced Peptide-PRISM, a method customized to identify ncORF peptides within the tumor immunopeptidome. Their study included diverse cancer types such as melanoma, lung cancer, glioblastoma, triple-negative breast cancer, and mantle cell lymphoma. They applied an FDR of 1% to retrieve putative HLA-bound peptides, which are provided in their supplementary table 3. Ruiz Cuevas *et al.* (*19*) integrated Ribo-Seq and MS to characterize the proteome and immunopeptidome of three human diffuse large B cell lymphomas bearing HLA A*01:01, A*02:01, A*02:06, A*31:01, B*08:01, B*15:01, B*44:02, B*51:01, C*03:01, C*07:01, C*07:04, and C*14:02. Only peptides with a sample-specific FDR of 1% were retained (entry PXD020620 in PRIDE). IEAtlas (*37*) is a comprehensive database that collected and reanalyzed publicly available MS-based HLA immunopeptidome datasets from 15 cancer types (acute myeloid leukemia, T and B cell acute lymphoblastic leukemia, breast cancer, chronic lymphocytic leukemia, chronic myelogenous leukemia, colon carcinoma, glioblastoma, kidney clear cell cancer, lung cancer, lymphoma, melanoma, meningioma, neuroblastoma, and ovarian cancer) and 30 noncancerous tissues. MaxQuant (84) was used to search against a curated database of noncanonical ORFs, applying an FDR of 5%.

### Analysis of MS immunopeptidomics data from HCC

We analyzed MS immunopeptidomics data from hepatocytes obtained from seven patients diagnosed with HCC, available from de Beijer *et al.* (*39*) (table S23). We built a curated database comprising the annotated human proteome sourced from Swiss-Prot/TrEMBL, including isoforms (comprising 103,789 sequences, downloaded on 21 April 2023), alongside a nonredundant compilation of tumor-associated ncORFs predicted with ribORF v1.0 (*29*) (5021 noncanonical sequences). We searched for significant matches with MHCquant (*83*), an nf-core pipeline implemented within Nextflow, specifically designed for quantitative processing of data-dependent acquisition peptidomics data. The search engine Comet (85), in conjunction with Percolator, was used for peptide identification, with default parameters and an FDR threshold set at 5% (table S26). We only considered uniquely matching peptides. We found evidence of peptides encoded by CTAs (12 cases) as well as ncORFs from tumor-specific lncRNAs (18 cases). All peptides derived from tumor-associated antigens (CTAs) reported by de Beijer *et al.* (*39*) except one (FPQSPLQGEEF in *MAGEC1*) were identified by our pipeline.

### Statistical analysis

Statistical analyses were performed using R (version 4.1.2). Comparisons between two distributions were performed using the paired Wilcoxon signed-rank test (Figs. 1B and 3B, and fig. S8) or the Kolmogorov-Smirnov test (Fig. 2E). The difference between two proportions was assessed using Fisher’s exact tests (Fig. 3E). After analyzing Gaussian distribution of data with the Shapiro test, Wilcoxon signed-rank test was performed to assess the differences between the MFI of the peptides with respect to the MFI of the peptide control (Fig. 4C). To test the immunogenicity of the ncORF peptides compared with their background signal, we performed paired two-sample *t* tests.
